# Finger Temperature: A Psychophysiological Assessment of the Attentional State

**DOI:** 10.3389/fnhum.2019.00066

**Published:** 2019-03-13

**Authors:** Rodrigo C. Vergara, Cristóbal Moënne-Loccoz, Camila Ávalos, José Egaña, Pedro E. Maldonado

**Affiliations:** ^1^Departmento de Neurociencia, Facultad de Medicina, Universidad de Chile, Santiago, Chile; ^2^Instituto de Neurociencia Biomédica, Facultad de Medicina, Universidad de Chile, Santiago, Chile; ^3^Departamento de Anestesiologiá y Medicina Perioperatoria, Facultad de Medicina, Universidad de Chile, Santiago, Chile

**Keywords:** EEG, autonomic nervous activity, attention, finger temperature, oscillations

## Abstract

Attention is a key cognitive phenomenon that is studied to understand cognitive disorders or even to estimate workloads to prevent accidents. Usually, it is studied using brain activity, even though it has many psychophysiological correlates. In the present study, we aim to evaluate if finger temperature, as a surrogate of peripheral vasoconstriction, can be used to obtain similar and complementary information to electroencephalography (EEG) brain activity measurements. To conduct this, 34 participants were recruited and submitted to performing four tasks—one as a baseline, and three attentional tasks. These three attentional tasks measured sustained attention, resilience to distractors, and attentional resources. During the tasks, the room, forehead, tympanic, and finger temperatures were measured. Furthermore, we included a 32-channel EEG recording. Our results showed a strong monotonic association between the finger temperature and the Alpha and Beta EEG spectral bands. When predicting attentional performance, the finger temperature was complementary to the EEG spectral measurements, through the prediction of aspects of attentional performance that had not been assessed by spectral EEG activity, or through the improvement of the model’s fit. We also found that during the baseline task (non-goal-oriented task), the spectral EEG activity has an inverted correlation, as compared to a goal-oriented task. Our current results suggest that the psychophysiological assessment of attention is complementary to classic EEG approach, while also having the advantage of easy implementation of analysis tools in environments of reducing control (workplaces, student classrooms).

## Introduction

Attention is a key cognitive phenomenon whose mechanism impacts a wide range of disciplines (Petersen and Posner, 2012; Langner and Eickhoff, 2013); from understanding and treating attention deficit hyperactivity disorder (ADHD) (Castellanos and Tannock, 2002; Huang-Pollock et al., 2012; Tamm et al., 2012) to examining its consequences during driving or industrial accidents (Hancock, 1986; Pilcher et al., 2002; Hancock and Vasmatzidis, 2003; Cheema and Patrick, 2012). Because of the consequences of assessing attention, especially in real-work environments, fast and reliable examination of this cognitive ability has become an essential goal in neuroscience. Typically, cognitive attention is assessed through brain activity (Petersen and Posner, 2012); however, attentional processes may exhibit many other physiological correlates (Lehrer et al., 2010; Werkle-bergner and Sommer, 2014; Liao et al., 2016; Binda and Gamlin, 2017; Vergara et al., 2017). Currently, some features of the cerebral electric activity (e.g., ERPs and spectral activity) are considered the best predictors of attentional states (Hogervorst et al., 2014; Kumar et al., 2018), but EEG presents major limitations when these measurements are applied in real working environments.

Attention assessed through EEG measurements are characteristically based on two major approaches; spectral activity and event-related potentials (ERPs). In general terms, oscillatory activity has been related to ongoing global states of attention and other cognitive functions. In contrast, the evoked potentials are associated with activities specifically elicited by a given stimulus. These latter responses were examined after averaging large amounts of stimulus-evoked brain activity trials, which were presumed to be similar enough to produce a meaningful average (Luck, 2005). Depending on the stimulus properties, specific evoked potentials have been reported in many studies (Hajcak et al., 2010; Ganushchak et al., 2011), particularly associated to attention—frequently studied for its relevance in attention deficit disorders (Johnstone et al., 2013).

Despite the useful information that can be obtained from ERPs, they are highly sensitive to stimulus properties, also they required a high number of repetitions of the same stimulus (Luck, 2005). For this reason, ERPs require highly controlled environments. Nonetheless, various methods have been used to assess single-trial evoked potentials (one stimulus evoked-brain-activity; Delorme et al., 2015; Williams et al., 2015); however, these methods are still under development (Li et al., 2018). While only the P300 evoked potential has been widely used as a single trial (Lin et al., 2018), but to our knowledge, the literature regarding its potential use in attention is still limited (Käthner et al., 2014).

In contrast, oscillatory activity is widely used in contexts of reduced control to estimate workload and fatigue (Chuang et al., 2018; Karthaus et al., 2018) as well as many other cognitive functions (Klimesch, 1999; Başar and Güntekin, 2008; Sauseng and Klimesch, 2008; Buzsáki and Watson, 2012; Zoefel and VanRullen, 2017). EEG oscillatory activity is the result of the rhythmic activity of large populations of neurons (Buzsáki et al., 2012), containing the most common spectral bands studied in attention; theta (4–8 Hz), alpha (8–13 Hz), and beta (14–30 Hz). Theta and especially the frontomedial theta has been shown growing more powerful during sustained attention tasks, yet this gain has traditionally been associated with deteriorated attention (Lal and Craig, 2002; Wascher et al., 2014). Moreover, theta is associated with performance improvement during non-fatiguing tasks (Missonnier et al., 2006; Ahveninen et al., 2013). On the other hand, alpha oscillations are thought to play a role in the inhibition of task-irrelevant processes, specifically, its power increments in sensory areas when not being used to drive the attentional process (Kelly et al., 2006; Anderson and Ding, 2011; for an alpha review: Klimesch, 2012). Beta has traditionally been associated with top-down control mechanisms; however, it may display similar inhibitory properties akin to alpha ones (van Ede et al., 2014). Yet others have proposed that beta relates to maintaining cognitive control during pauses within an attention task (Shapiro et al., 2017). All these three bands have been used to build “attentional indices” (Molteni et al., 2007; Coelli et al., 2015, 2018).

Despite substantial evidence in favor of utilizing EEG features to assess the attentional processes, this approach has its shortcomings. EEG activity can be assessed under uncontrolled environments but is technically complex, sensitive to muscular and electric noise, generally uncomfortable, with high computational demands. Moreover, highly noisy recordings can produce high classification results due to overfitting, when combining machine learning techniques with small sample sizes. Even when using larger sample sizes, the differences in subjects may contribute to different EEG activities evoked by the same stimuli (Hogervorst et al., 2014).

Since attentional processes are a global brain state, their manifestations should not only be revealed as changes in brain activity but also through other psychophysiological markers. Indeed, it is known that pupillary dilation (Hoeks and Levelt, 1993; Liao et al., 2016; Binda and Gamlin, 2017), oculomotor activity (Werkle-bergner and Sommer, 2014; Meyberg et al., 2017; Schwedhelm et al., 2017), heart rate variability (Lehrer et al., 2010), and peripheral vasoconstriction are associated to attentional performance (Vergara et al., 2017). Despite their potential usefulness, along with EEG measures, psychophysiological activity is seldom used in the practical assessments of attention. For instance, neurofeedback that is used for ADHD and other attentional disorders is done only with brain-related variables (Ordikhani-Seyedlar et al., 2016). Similarly, EEG spectral activity alone is considered the gold standard in predicting alert/workload/fatigue to prevent accidents (Kumar et al., 2018).

We conjecture that psychophysiological correlates such as electrodermal response, heart rate variability, or peripheral vasoconstriction, provide similar and/or complementary information, as compared to brain-related variables solely, which can be potentially employed in real therapies and interventions or working environments to reduce health risks. In this work, we aim to evaluate the association of classic attentional, brain-related measurements with the general psychophysiological response, triggered by attentional demands. Specifically, we hypothesize that EEG spectral activity and peripheral vasoconstriction, measured through skin temperature change, can complement each other to predict attentional performance.

## Materials and Methods

### Participants

A total of 34 participants were recruited (18 females and 16 males) of ages 19 to 36 years old, with a mean age of 25.17 ± 4.8 years (mean ± SD). All volunteers provided written informed consent, following the Declaration of Helsinki, to participate in this study (approved by the Comité de Ética de Investigación en Seres Humanos from the Facultad de Medicina, Universidad de Chile), project number ID 060-2015, ACTA AP-65. This study is partially based on a previously conducted one (Vergara et al., 2017). The current study can be differentiated from the previous one as it includes EEG measurements as well as adds 15 more subjects to the sample size.

### Tasks

Participants performed the same tasks in similar conditions, as reported in Vergara et al. (2017) in their study. All the participants briefly executed the following four tasks: The baseline task (BT), the continuous performance task (CPT), the flanker task (FT), and the counting task (CT). The BT was to measure the baseline variation of body temperatures during a resting performance. The remaining tasks were designed to measure the specific attentional features; CPT tested for sustained attention, FT tested for resilience to distractors, and CT for attentional resources. All tasks last approximately 10 min.

#### Continuous Performance Task (CPT)

**FIGURE 1:**
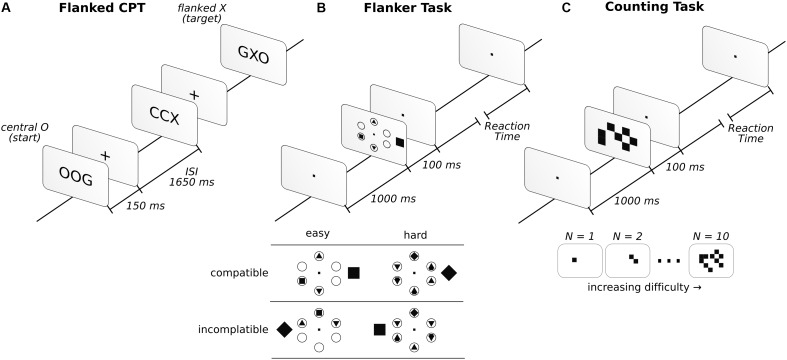
Description of the three attentional tasks in the order in which they were performed. Before starting the attentional tasks, a baseline was taken with the instruction to “sit and relax” (see “Materials and Methods” for more details). In the flanked CPT **(A)**, participants had to report whether the sequence of central letters, O-any letter-X, appeared. In the FT **(B)**, participants had to report a square or a diamond inside one of six circles. If the inside target figure (square or diamond) matched the external figure, presented outside the circles, it was considered compatible, while those that did not match were incompatible. In the CT **(C)**, participants had to report the number of squares observed.

This task consisted of the detection of a sequence of target letters with a low frequency of appearance (approximately 15%). To increase the difficulty of the task, we flanked the target letter with an additional letter to the right and left of the central letter. As such, we displayed three letters at the same time for 150 ms, followed by a fixation cross for 1,650 ms. If the central letter was X and, two trials back, the central letter was O, participants reported it by pressing a button (go condition). Reports of seeing this sequence when it was absent were considered false alarms (false alarm condition). A description of the task can be found in Figure 1A. The go condition was randomly displayed in 15% of a total of 400 trials, and the letters employed for the displays included C, G, O, Q, H, and X. All participants had to detect the same letter sequence.

#### Flanker Task (FT)

The task was configured following Green and Bavelier (2003). In this task, six circles were arranged equidistantly apart (2.1°) from the center of the screen, in a circular fashion. In each trial, a distractor outside the circular array was presented. It could be shaped as a square or a diamond and was presented 0.5° to the left or right (inside the array) or 4.2° (outside the array) from the center of the circular array. When the shape was presented inside the circular array, it was at 0.3° or at 0.9° from central fixation when presented inside or outside, respectively. The subjects were asked to report whether a square or diamond was present inside the circles. The other circles were either blank or filled with different geometric shapes, such as triangles and circles (see Figure 1B for example). The diamonds and squares were never displayed inside the circles, simultaneously, in the same trial. However, four (easy condition) or six (hard condition) shape-filled circles were evenly distributed over 320 trials. Additionally, to counter this, half of the trials were compatible (the figure outside the circular array matched the target shape inside), while the other half were incompatible (the figure outside the circular array did not match the target shape inside). The array was presented for 100 ms, followed by an unlimited amount of time to answer. The participants had to answer to continue with the task. Once the answer was provided, a 1,000 ms fixation dot was displayed. Descriptions of the task and conditions can be found in Figure 1B. For additional details regarding the task, see Proksch and Bavelier (2002) and Green and Bavelier (2003).

#### Counting Task (CT)

The task was implemented as described by Green and Bavelier (2003). In this task, a random number of squares was presented on the screen. The participants had to report the number of squares that they observed. The squares (0.5° × 0.5°) were randomly displayed over a 10° × 10° square array centered on the screen (Figure 1C). The set of squares were displayed for 100 ms, followed a visual fixation dot while awaiting the participant’s answer. The participants needed to respond to continue the task. Once a response was given, the fixation dot was displayed for 1,000 ms before the next trial. The task difficulty was modulated by presenting any number of squares, from 1 to 10, in an even distribution across 200 trials.

In our previous report (Vergara et al., 2017), we presented the tasks in the fixed order: BT, CPT, FT, and then CT. Therefore, the tasks of the previous 19 participants are present in that order. The 15 new participants included in this study were assigned the tasks in all possible orders. We included new orders to examine whether the respective order was relevant to any of the reported effects. To avoid thermal noise in our measurements, the environmental temperature was kept constant for each subject. The temperature between the subjects was homogeneously selected to be within a range of 18–26°C. This range was chosen based on previous room/environmental temperature studies (Steinmetz and Mussweiler, 2011; Cheema and Patrick, 2012; Huang et al., 2014; Schilder et al., 2014). Finger and environmental temperatures were measured using two Dallas DS18B20 thermometers, and an Arduino UNO board. One thermometer was positioned at the tip of the left ring finger, and the other was positioned at 40 cm to the front, 20 cm to the left, and it was lifted from the desk by 5 cm in front of the participant.

### EEG Recordings and Signal Processing

During the experiment, we recorded 32 channels of continuous EEG signals, using a Biosemi ActiveTwo System in an extended 10–20 configuration (Jasper, 1958). Two electrodes were placed above and below the right eye and two were placed on the external canthi of both eyes; this configuration was used to record the vertical and horizontal electrooculogram (EOG), respectively. The data was recorded at 2,048 Hz, re-referenced to a common average reference and band-pass filtered between 1 and 40 Hz using a symmetric linear-phase FIR filter of firwin design with a Hamming window (acausal, zero-phase delay, and applied in one-pass forward). The filter length was 3.3 s. The width of the transition band of the filter was 1 Hz at the low cut-off frequency and 10 Hz in the high cut-off frequency. The pass-band ripple of the filter was 0.0194 dB and the stop-band attenuation was 53 dB.

Data segments containing muscle movements and other non-eye blinks recorded artifacts were eliminated through the automatic, peak-to-peak threshold within a windows of 2 s. Subsequently, continuous data was submitted to the independent component analysis (ICA) to remove vertical and horizontal EOG components of the signals. Following this, continuous EEG epoching was conducted for each frequency band into a contiguous duration window, corresponding to 10 cycles of the central frequency of the band. Thus, the duration of the theta (4–8 Hz), alpha (8–13 Hz), and beta (14–30 Hz) epochs were of 1.8, 1.0, and 0.45 s, correspondingly. The artifact detection was performed on epoched data, using a peak-to-peak threshold algorithm with a voltage threshold of 250 μV. All the epochs containing the detected artifacts were rejected. The power spectral density (PSD) was obtained for each frequency band’s epoch, through a multitaper spectrum estimation, using multiple DPSS tapers with a half-bandwidth window of 4 Hz. The PSD values were then transformed to decibels (dB) by applying a natural logarithm and then multiplying by 10. For the data analysis we selected middle-line electrodes, which have previously been related to attention (Borghini et al., 2014; Hogervorst et al., 2014) for all the aspects of our tasks; sustained attention (Kelly et al., 2003), resilience to distractors (Bonnefond and Jensen, 2012), working memory (Freunberger et al., 2011), and attentional resources (Ghorashi and Spencer, 2015).

### Data Analysis

The analysis was performed to evaluate two main questions: Does finger temperature behave similarly to EEG spectral activity during the tasks? And, does finger temperature predict attentional performance as well as the EEG spectral activity? For the first question, to compare the temporal dynamics of temperature and EEG spectral activity regardless of the time that a participant needs to complete a task, we scaled the time so that all tasks last the same duration for each participant. This step is critical to compare task dynamics rather than time dynamics. If this no escalation wouldn’t be done, we would need to truncate the participants that lasted longer. Specifically, we calculated a representative normalized timeline for each task/instruction, which starts at 0 and ends in 1 (in this scale 0.5 would equate to half of the task). To fit the temporal data of each participant in this timeline without losing temporal resolution, we estimated a reference duration for each task/instruction by calculating the average time taken by the group of participants in seconds. In this way, we determine the number of points between 0 and 1 of the normalized time scale in granularity of seconds (e.g., average duration of 600 s for a task means 600 points between 0 and 1 in the normalized timeline of the task). Finally, each participant’s task related temperature dynamic was then linearly interpolated within that common space. This scaling allow us to compensate the time spent on the task through inter-subject variability, keeping the duration of each task proportional (i.e., each participant start at 0 and ends at 1). Importantly, this normalization does not affect importantly the data considering that all tasks last approximately the same. To control for environmental temperature differences, we average centered each subject using the following procedure:

(1) $$T_{\mathrm{ij}}^{\text{o}}=(x_{\mathrm{ij}}-x_{j})+x_{g}$$

Where *x_ij_* is an individual observation *i*, of the temperature of a particular participant *j*, *x_j_* is the participant’s average temperature, *x_g_* is the grand average temperature for all participants, and *T°_ij_* is the centered temperature for observation *i*, and participant *j*. Once all the participants were normalized, we averaged their finger temperature and their theta, alpha, and beta spectral activities. Finally, we performed Spearman correlations to evaluate if the finger temperature displayed similar behavior to those of the spectral activity. To address whether finger temperature predicts attentional performance as well as EEG spectral activity, we employed the changes in finger temperature and spectral activity, from the beginning and the end of each task, to predict attentional performance. Following the procedure of Vergara et al. (2017), we used ΔFingerT° defined as:

(2) ΔFingerT°=Final Finger Temperature−Initial Finger Temperature

Where, the final and initial finger temperature is approximately the average of 1 min of it, obtained from the fingertip thermometer, at the end or the beginning of one of the four tasks, respectively. The Initial finger temperature did not include the instruction time and was restricted to the beginning of the task. We used the same approach for spectral activity, replacing finger temperature with the EEG spectral power, obtained at the first and last minute of each task, leading to 12 new variables: ΔTheta, ΔAlpha, ΔBeta, each in four midline EEG electrodes (Oz, Pz, Cz, and Fz). To predict attentional performance, we used multiple linear regression models, using ΔFingerT°, ΔTheta, ΔAlpha, ΔBeta for each electrode as predictors.

Since all 12 EEG spectral variables are likely to be correlated, we evaluate the collinearity using the variance inflation factor (VIF) (García et al., 2015). Linear models presenting VIFs higher than three were then submitted to a dimension reduction, to reduce the collinearity and the number of variables used as predictors. The dimension reduction, in our case, was aimed to average those variables which were consistently associated, so it is possible to assume that they measure the same phenomenon. This allowed us to reduce the 12 spectra EEG variables into fewer independent variables, where they were grouped based on their covariances. This procedure is widely used for this purpose (Gaskin and Happell, 2014). In doing so, we performed a factor analysis using maximum likelihood as an extraction method, with varimax rotation (intending to reduce collinearity), where the number of factors to extract was decided based on the Eigenvalue > 1 criteria (Raîche et al., 2013). The variables that loaded the same factor were then averaged. In case of detecting cross loaders in the factor solution (variables loading over 0.3 in more than one factor), the averages were built using the highest loading and ignoring the lowest. Before averaging the variables, we evaluated the internal consistency of each dimension using Cronbach’s alpha (Cronbach, 1951). These averages were then used in the regression models. If the linear models with reduced dimensions presented a VIF higher than three, then they were revised by checking each regressor separately, and then the combinations among them, leaving behind the regressors that explained more variance (squared-R based), thus, keeping the VIF below 3. The dependent variables were different depending on the task, and also some new independent variables which were included according to each case. For the CPT we used go accuracy and reaction times as dependent variables, without including any new independent variables. For FT we used incompatible minus compatible reaction times as the dependent variable, following previous reports (Proksch and Bavelier, 2002; Green and Bavelier, 2003). As an independent variable (predictor) we included the difficulty (easy or hard). Finally, for the CT we used the error ratio as the dependent variable, and the number of squares presented in the task as a predictor (independent variable). We also reported whether the data passed the assumption of normality of the dependent variable and the residuals, as well as the homoscedasticity. The homoscedasticity (homogeneity of variance) was evaluated using the non-constant variance score test, and for normality, we used the Shapiro–Wilk test. In this case, we reported the normalized regression coefficients to be comparable to the predictor’s coefficients of EEG spectral activity with ΔFingerT°. Finally, to evaluate the effects of the order in which the tasks were presented, we performed a one-way ANOVA, to evaluate if all the dependent variables, previously described, were different due to the task order. Given the ordinal nature of this variables and its tight relation to the duration of the experimental session, we also included as a covariable in the multiple linear regression models.

### Software

For every statistical analysis, we used the R-project (R Core Team, 2015), using the following packages: Lattice (Sarkar, 2008), ez (Lawrence, 2016), car (Fox and Weisberg, 2011), psych (Revelle, 2016), nFactors (Raiche, 2010), GPArotation (Bernaards and Jennrich, 2005), and MVN (Korkmaz et al., 2014). The processing of data processing and figures were developed with Python 2.7.1, Anaconda 2.4.1 (Python Core Team, 2015), using the following packages: Pandas (McKinney, 2010), MNE (Gramfort, 2013; Gramfort et al., 2014), and Matplotlib (Hunter, 2007). The EEG data processing was carried out through the MNE-python software (Gramfort, 2013).

## Results

### Finger Temperature and Spectral Activity Association

**FIGURE 2:**
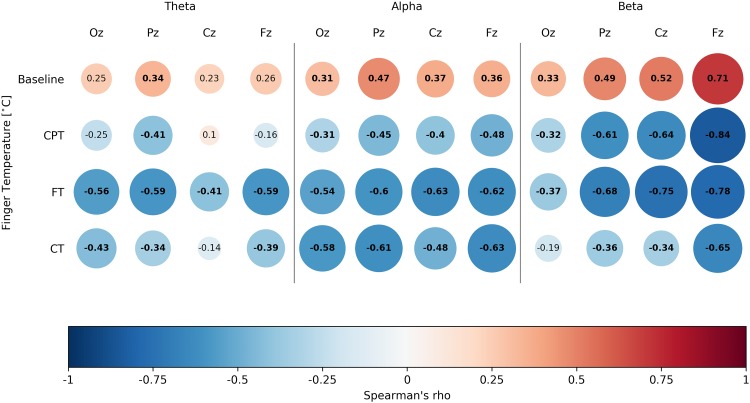
Spearman’s correlations presenting the association between finger temperature and EEG spectral activity for each of the four tasks of the study. Presents Spearman’s correlations of all three tested spectral bands. Each box represents a correlation test, with Spearman’s rho inside it. Colors are assigned for an easy reading, where blank spaces denote non-significant correlations (we considered significant results with *p* < 0.001, based on Bonferroni’s correction).

**FIGURE 3:**
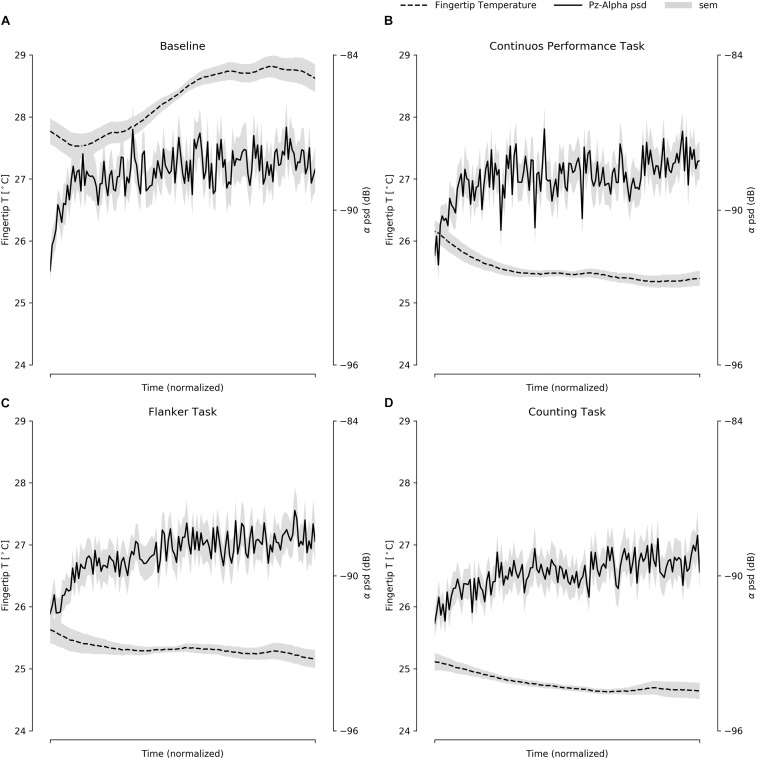
The average of the fingertip temperature and EEG spectral alpha band activity, presented within the normalized time for all four tasks: **(A)** BT, **(B)** CPT, **(C)** FT, and **(D)** CT. In gray, the standard deviation of the measures are presented.

To assess whether finger temperature measurements for the four different tasks behaved similarly to the EEG spectral activity, we ran a set of correlations using the grand averaged EEG spectral activity and finger temperature. Even when using a conservative approach for these correlations, such as a Bonferroni’s multiple comparison correction with an alpha value of *p* < 0.001, most of them were significant (Figure 2). In general terms, the cognitive tasks presented a consistent inverse association of spectral activity with the finger temperature, while for the baseline they presented a positive association. The beta and alpha bands were more consistently associated to finger temperature changes, where only the alpha band displayed a remarkably similar curve in the function of task time (Figure 3B). However, it is critical to notice that the EEG presented a noisy dynamic, as compared to the finger temperature, while the latter presented a slightly slower dynamic throughout the duration. Overall, these results support that finger temperatures are associated with the classic brain attentional measurements, related to attentional performance. Interestingly, this relation is inverse for goal-directed tasks (CPT, FT, CT), in contrast with the BT, which presented a direct association (Figure 3).

### Collinearity and Dimension Reduction

Once we have already described a population relationship between the EEG spectral activity and finger temperature, we wanted to evaluate if, at the participant level, we were able to predict the attentional performance, based in spectral activity and finger temperature. Therefore, we used the difference between the ending and the beginning of each cognitive task as predictors (for further details, see the section “Materials and Methods”). Given the high correlation presented among the different spectral bands and electrodes, where the signals were measured, we violated the independence of multiple linear regression model assumptions by garnering a high collinearity (VIF > 20, ref < 3). Thus, we submitted all the deltas of the spectral activities for a dimension reduction, for each task, using factor analysis. For the CPT, we detected three dimensions; one related exclusively to ΔTheta, another to all the ΔAlpha electrodes, but also including the Oz ΔBeta, and finally the ΔBeta for the Pz, Cz, and Fz electrodes (Table 1). All the three factors presented high internal consistencies: The theta band Cronbach’s alpha = 0.9; the alpha band plus the occipital beta presented a Cronbach’s alpha = 0.93, and the parieto-center-frontal beta band presented a Cronbach’s alpha = 0.89 (Table 1). For the FT, we found three factors, one associated to ΔTheta, another to ΔAlpha, and the last one to ΔBeta. Their internal consistencies, measured through the Cronbach’s alpha were, 089, 0.94, and 0.92, respectively. Finally, for the CT, only two factors were found, one associated with ΔTheta, and another to ΔAlpha and ΔBeta. The Cronbach’s alpha values for these two factors were 0.89 and 0.94, respectively. All the results are summarized in Table 1. Given the current results, the differences were average agglomerated using each factor as reference, depending on the results for each task, to then use these averages as predictors in the linear regression models. In case of having cross loaders in the factor solution (variables loading over 0.3 in more than one factor), the averages were built using the highest loading and ignoring the lowest (Table 1, marked in bold). From this point on, we use 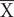
Δ to denote the spectral activity deltas that were then averaged, based on the factor analysis results.

**Table 1:** Factor analyses and internal consistencies results for all tasks.

Spectral activity	Electrode	CPT			FT			CT	
		Factor1	Factor2	Factor3	Factor1	Factor2	Factor3	Factor1	Factor2
ΔTheta	*Oz*	0.55	**0.67**		**0.77**				**0.87**
	*Pz*		**0.71**		**0.57**				**0.7**
	*Cz*		**0.81**		**0.87**				**0.67**
	*Fz*		**0.88**		**0.81**				**0.84**
ΔAlpha	*Oz*	**0.96**					**0.98**	**0.91**	
	*Pz*	**0.61**			0.58		**0.64**	**0.78**	
	*Cz*	**0.73**			0.59		**0.68**	**0.78**	0.41
	*Fz*	**0.78**		0.44	0.57		**0.62**	**0.79**	0.47
ΔBeta	*Oz*	**0.64**				**0.72**	0.47	**0.7**	
	*Pz*			**0.74**		**0.89**		**0.77**	
	*Cz*			**0.86**		**0.92**		**0.66**	
	*Fz*			**0.91**		**0.83**		**0.57**	
Internal consistencies	Cronbach’s alpha	0.9	0.93	0.89	0.89	0.94	0.92	0.89	0.94

*Bold loadings indicate the variables used for averaging. Internal consistencies are indicated at the bottom of the table for each factor, including only the bold loadings.*

### Multiple Linear Regressions

For the CPT, we were able to significantly estimate the reaction times for go condition, using ΔFingerT° [*F*(1.28) = 11.62, *p* = 0.0019, *R*^2^ = 0.293; β_ΔFingerT°_ = 0.54, *t* = 3.40, *p* = 0.0019; homoscedasticity: χ^2^ = 0.37, df = 1, *p* = 0.53; residual normality: *W* = 0.96, *p* = 0.45, dependent variable normality: *W* = 0.949, *p* = 0.087]. The spectral EEG activity variables were not significant predictors of the CPT go reaction times; however, the 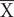
ΔBeta average and the order of the task presented a significant interaction (β_
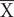
ΔBeta_ = 0.32, *t* = 0.92, *p* = 0.36; β_TasksOrder_ = 0.37, *t* = 1.70, *p* = 0.09; βInteraction = −0.98, *t* = −2.57, *p* = 0.016), when predicting the CPT accuracy [*F*(3,27) = 5.57; *p* = 0.004, adjusted-*R*^2^ = 0.313; homoscedasticity: χ^2^ = 2.64, df = 1, *p* = 0.103; residual normality: *W* = 0.929, *p* = 0.043, dependent variable normality: *W* = 0.627, *p* = 1.017E-08]. For accuracy, the ΔFingerT° was not a significant predictor. As such, for a sustained attention task, the finger temperature was able to predict the distinct aspect of attentional performance, compared to the EEG spectral activity, suggesting that despite the association between both (Figure 2), they provide complementary information. For the FT, only the spectral activity was a significant predictor, concretely the FT 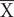
ΔAlpha [*F*(1,66) = 8.52; *p* = 0.004, *R*^2^ = 0.114; β_
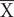
ΔAlpha_ = −0.33, *t* = −2.91, *p* = 0.004; homoscedasticity: χ^2^ = 1.08, df = 1, *p* = 0.29; normality: *W* = 0.977, *p* = 0.27, dependent variable normality: *W* = 0.983, *p* = 0.39]. These results suggest that finger temperature modulations are not associated to this specific feature of attention, which can be captured by the EEG alpha spectral band. Finally, for the CT we found a complementary situation, where the ΔFingerT° (β = −0.104, *t* = −3.140, *p* = 0.0018), CT 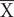
ΔAlphaBeta (β = 0.228, *t* = 6.83, *p* = 4.55E-11), and the number of squares presented were significant predictors of the error ratio in the task (β = 0.779, *t* = 23.45, *p* = 2E-16). These results demonstrate that finger temperature served as a complement to the spectral EEG activity when predicting attentional performance in this task, thereby, explaining 66% of the variance [*F*(3,302) = 201, *p* = 2.2E-16, adjusted *R*^2^ = 0.663; highest VIF = 1.0097; homoscedasticity: χ^2^ = 53.41, df = 1, *p* = 2.7E-13; normality: *W* = 0.966, *p* = 1.3E-06, dependent variable normality: *W* = 0.864, *p* = 2.2E-16]. In order to evaluate the relative weight of the model fit of the EEG and finger temperature variables, we explored the reduction in it when removing the 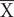
ΔAlphaBeta or the ΔFingerT°. We observed a reduction of 0.05 in the adjusted-*R*^2^ when removing the 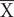
ΔAlphaBeta and of 0.01 for the ΔFingerT°. This means that the 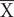
ΔAlphaBeta explains around 5% and the ΔFingerT° about 1% of the model’s variance. This means that the number of squares explains over 60% of the variance in the error ratio made by the subject. Beyond the experimental condition, the EEG measurements explain more variance than the finger temperatures. Overall, the results presented here support that the classic brain-related variables are capable of being complemented with a psychophysiological approach. Concretely, as summarized in Table 2, the EEG related variables were unable to capture the performances predicted by the finger temperature and vice versa in the case of CPT and FT.

**Table 2:** Summary of all linear regression models results.

Tasks	CPT		FT	CT
Performance	*Accuracy*	*Reaction times*	*Reaction time diff.*	*Error*
Δ Finger	n.s.	0.54^∗∗^	n.s.	−0.10^∗∗^
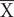 ΔTheta	n.s.	n.s.	n.s.	n.s.
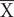 ΔAlpha	n.s.	n.s.	−0.33^∗∗^	n.s.
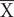 ΔBeta	0.32	n.s.	n.s.	n.s.
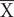 ΔAlphaBeta	n.s.	n.s.	n.s.	0.22^∗∗∗^
Order	0.37	n.s.	n.s.	n.s.
Order^∗^DeltaBeta	−0.98^∗^	n.s.	n.s.	n.s.
Square N°				0.77^∗∗∗^
*R*^2^	0.313	0.293	0.114	0.663

*Each table’s column represent one linear regression model. Standardized coefficients are presented. Non-significant results were included as n.s., and therefore pruned from the model. In case of interaction, main effects are left to improve interpretability. Square Number were only tested in CT, therefore, left in blank for the other tasks. Accuracy in CPT refers to the number of correct answers in Go condition. Reaction times are those of the correct answers in Go condition. Reaction time difference in FT is the difference between incompatible and compatible condition’s reaction times. Finally, error refers to the number of wrong answers given in counting task. Significances are stated as follows: ^∗^p < 0.05, ^∗∗^p < 0.01, ^∗∗∗^p < 0.001.*

## Discussion

The results of our experimental design can be summarized in two major statements: First, the finger temperature might not measure the same physiological processes as the EEG spectral activity. Second, these variables are rather complementary when trying to estimate attentional performance. In the following sections, we address the consistency of our spectral EEG findings with the previous literature, followed by how we interpret the association of finger temperatures. Finally, we address the possible applications of finger temperatures for research regarding attention, accident prevention, or workload and fatigue prediction.

### Spectral EEG Signals and Attention

In this work, we first examined the three different EEG frequency bands and the finger temperature to predict attentional performance. We found that theta was not a significant predictor of performance in any of the three attentional tasks used (Table 2). The finding contests previous reports showing that theta oscillations had been ascribed to numerous cognitive processes, such as working memory (Raghavachari et al., 2001), expectation (Rungratsameetaweemana et al., 2018), or attention (Fries, 2015). During our tasks, most of these cognitive abilities are deployed to fulfill the tasks. Thus it is intriguing that the theta band activity exhibited a weak correlation with the ΔFingerT° and was not a good predictor of attentional performance. In contrast, the alpha activity consistently correlated with the ΔFingerT° across all tasks and locations (Figure 2). The alpha band activity was the only significant predictor of FT performance. These results appear to be supported by a more robust association between this frequency band and the attentional process it underlies, which is related to the inhibition of non-relevant areas (Fu et al., 2001; Jokisch and Jensen, 2007). Finally, beta activity resulted in the strongest correlation with the finger temperature. Also, it was a significant predictor of CPT performance. This result is consistent with its putative, top-down control over cognitive processes. Our tasks were selected in part because they required various abilities, but also because all of them could be considered to depend on the activation of a high-order phenomena, requiring top-down control mechanisms.

As stated before, EEG is likely to be the most non-invasive tool used to assess and explain the subjects’ performance in the different attentional tasks, but it should be considered that most of the reports about their role in brain processes are the result of complex experimental settings. Furthermore, EEG signals have to be subjected to intricate analysis algorithms that modify the original signal in various forms.

### Finger Temperature and Attention

To our knowledge, there are limited articles testing the peripheral temperatures and its relation to attention. Nonetheless, the fact that we extended the sample and included the EEG measurements of Vergara et al. (2017) means that we extended sample size using the same experimental design. This allowed us to directly contrast our previous results to evaluate the robustness of peripheral temperature as a marker of attention. In our present study, finger temperature was a significant predictor of attentional performance for two of the three tasks performed, the FT being the one where the finger temperature was not a significant predictor. This contrasts the previous results, where the finger temperature was used to estimate the attentional performance of all three tasks (Vergara et al., 2017). We obtained a significant *p*-value in our previous report; however, given the sample size used in that study, it was possible to obtain non-reproducible results (Nuzzo, 2014). Simultaneously, it is interesting that we were able to obtain similar results for two of the three tasks, supporting that finger temperature can be used as an attentional performance marker. If the tasks are further examined, where the finger temperature was a significant predictor, it will be found that they target particular features of attention. CPT is associated with sustained attention (Riccio, 2002; Huang-Pollock et al., 2012), while FT was developed to measure resilience to distractors (Proksch and Bavelier, 2002), and CT is related to attentional resources (Green and Bavelier, 2003). Altogether, we interpret that a decrement in finger temperature is associated with an increment in arousal, which will help sustain an attentional state, as well as recruit more resources, but not necessarily improve the ability to discriminate between the target and the distractors when solving the task.

Then, why would finger temperature be associated with arousal? It is known that many, some of them autonomic, psychophysiological mechanisms will be triggered into coupling with environmental demands (Jansen et al., 1995). Probably, the most likely scenario of this coupling is during the fight or flight response (FOF) (Cannon, 1929). In this scenario, a highly salient stimulus from the environment triggers many psychophysiological mechanisms to evaluate the danger to then decide if the response should be FOF.

The FOF system increases arousal to cope with stressful situations (Kemeny, 2003) where the heart rate (Kreibig, 2010) and peripheral vasoconstriction (Ulrich-Lai and Herman, 2009), among others physiological responses, appear. We may consider that the FOF response is an acute version of a general physiological system triggered to cope with external demands. For instance, thermal stress induces vasoconstriction/dilation (Charkoudian, 2003; Johnson and Kellogg, 2010), which changes hand and feet temperatures (Cheung, 2015), but cognitive-demanding tasks also produce vasoconstriction (Iani et al., 2004) and can be related to subjective, self-reported stress (non-thermal stress) (Yamakoshi et al., 2007). We conceived that the set of physiological responses that appear in a triggered FOF response are proportional to the perceived stress/potential risk. Therefore, cognitive tasks will trigger a FOF-like response, according to the perceived stress/cognitive demand that the task represents. This claim would also be supported by Figure 2, were correlations between spectral bands and finger temperatures change direction in BT, as compared to goal-oriented tasks. We interpret this difference as a process where during BT, no external demand is imposed upon the participant and therefore, there is no trigger of this psychophysiological mechanism. Even though FOF is classically seen as an emotional response, it is necessarily related to attentional resource allocation and reorienting. The heart rate variability and peripheral vasoconstriction are already reported to be associated with attentional performance (Lehrer et al., 2010; Vergara et al., 2017). In summary, these results can be interpreted within a psychophysiological phenomenon that leads to an increase in arousal and attentional resources, which in turn is triggered by external demands and observed by modulations in autonomic responses, such as the peripheral body temperature.

### Differences Between Finger Temperature and EEG Spectral Activity

Finger temperature has proven to be useful in predicting attentional performance (Vergara et al., 2017). More importantly, finger temperature seems to complement spectral measurements. For instance, CPT reaction times were predicted by finger temperature but not by spectral activity, while accuracy was predicted only by spectral activity. Only in the case of CT we found that both were significant predictors. If we consider that spectral activity presents a significant correlation with finger temperature, how is it possible that ΔFingerT° is independent from 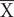
ΔAlphaBeta? (reflected in the highest VIF of 1.009; ref < 3). If we examine Figure 3, we will notice that despite the similarity in the dynamic of both curves, the spectral activity is noisier compared to the finger temperature. Also, it presents a faster dynamic, hence the use of Spearman’s monotonic correlation instead of the usual linear Pearson correlation. Given that we were using the difference between the first and the last minute of the task, the slower dynamic of finger temperature helps to increase the ΔFingerT°, as compared to spectral activity which rapidly plateaus. Also, the noisy EEG signal increased the variability of the difference between the last and first minute. It is most likely that the noise was due to artifacts such as fast fluctuations in impedance values, muscle contraction, eye movements or others. These differences between both the signals implied that when estimating the differences between the last and first minute, they resulted in independent variables. This is most interesting, considering that for CPT—a vigilant task—the finger temperature was a predictor of reaction times, while the spectral activity was of accuracy. We speculate that the differences between the dynamic of these two signals might be reflect different attentional processes. Naturally, a better suited experiment to disentangle each other’s roles would be required to understand the mechanisms underlying the autonomic and cortical neural correlates of attention. In the specific context of this article, we conclude that the EEG spectral activity and finger temperatures are robust predictors of sustained attention and attentional resources. For resiliency to distractors, the spectral activity seems to be a better predictor. Therefore, we also conclude that the EEG spectral activity can be complemented by the finger temperature when predicting attentional performance in tasks requiring long periods of attention, and when more resources are needed to fulfill a task.

One final interesting finding is the correlation between the finger temperature and the change in direction of the spectral activity during BT, when compared to all the performed three tasks (Figure 2). Only for BT the correlations are positive, while for all goal-oriented tasks this association is negative. The critical difference between these tasks is that BT is not goal oriented, or at least it does not have a clear expected outcome or external demand. The instruction is to relax, but no evaluation or answer is required during the task. As such, it is plausible that finger temperature, jointly with EEG spectral bands, can be used to discriminate between states where people are engaged in a goal-oriented task or are distracted toward a non-goal-oriented task (e.g., attending to their own thoughts). Our experimental design does not allow for an evaluation of this, however, it seems promising to use the association of EEG and finger temperatures as markers of task engagement.

### Possible Applications for Brain-Autonomic Joint Assessment of Attention

Our current results suggest two possible applications for joint, brain-autonomic assessment of attention. First, our results support the idea that the best way to estimate the attentional state of a person and avoid possible workplace accidents due to inattentive or drowsiness, is using a joint, brain-autonomic assessment of attention. However, this concept still requires additional research to determine the relative value of autonomic and brain-related measures, and the impact of non-regulated environments over these measures and their predictive power. Moreover, in this study, we used a simple approach (multiple linear regressions) which can be used as starting point to develop more accurate models using techniques such as random forest, support vector machine, artificial neural networks, or others. The fact that a simple approach proves to be enough to find the association between finger temperatures, EEG spectral activities, and attentional performance, is a good starting point to improve predictive models and, above all, include peripheral measurements as complements of EEG. In addition, these measures may have a role in predicting other cognitive outcomes, such as neurofeedback therapies, widely applied to ADHD (Ordikhani-Seyedlar et al., 2016). Overall our results contribute to the assessment of attention by suggesting a joint, brain-autonomic strategy, improving the prediction of the attentional state. This can be employed in the workplace environment and thus contribute to enhancing safety and mental health conditions.

## Author Contributions

The experimental design was developed by RV and PM. The data collection was performed by RV and CÁ. The analysis was conducted by CM-L and RV. All the electrical *ad hoc* devices and software were developed by RV. The manuscript was written by RV, CM-L, CÁ, JE, and PM.

## Conflict of Interest Statement

The authors declare that the research was conducted in the absence of any commercial or financial relationships that could be construed as a potential conflict of interest.
